# Ovarian metastases resection from extragenital primary sites: outcome and prognostic factor analysis of 147 patients

**DOI:** 10.1186/1471-2407-12-278

**Published:** 2012-07-03

**Authors:** Wenhua Li, Huaying Wang, Jian Wang, Fangfang LV, Xiaodong Zhu, Zhonghua Wang

**Affiliations:** 1 Department of Medical Oncology, Cancer Hospital of Fudan University, Shanghai Medical College, Shanghai, P. R. China; 2 Department of Gynecologic Oncology, Cancer Hospital of Fudan University, Shanghai Medical College, Shanghai, P. R. China; 3 Department of Pathology, Cancer Hospital of Fudan University, Shanghai Medical College, Shanghai, P. R. China; 4 Department of Medical Oncology, Cancer Hospital of Fudan University, Shanghai Medical College, No. 270 Dongan Road, 200032, Shanghai, China

## Abstract

**Background:**

To explore the outcomes and prognostic factors of ovarian metastasectomy intervention on overall survival from extragenital primary cancer.

**Methods:**

Patients with ovarian metastases from extragenital primary cancer confirmed by laparotomy surgery and ovarian metastases resection were retrospectively collected in a single institution during an 8-year period. A total of 147 cases were identified and primary tumor sites were colorectal region (49.0%), gastric (40.8%), breast (8.2%), biliary duct (1.4%) and liver (0.7%). The pathological and clinical features were evaluated. Patients’ outcome with different primary tumor sites and predictive factors for overall survival were also investigated by univariate and multivariate analysis.

**Results:**

Metachronous ovarian metastasis occurred in 92 (62.6%) and synchronous in 55 (37.4%) patients. Combined metastases occurred in 40 (27.2%). Bilateral metastasis was found in 97 (66%) patients. The median ovarian metastasis tumor size was 9 cm. There were 39 (26.5%) patients with massive ascites ≥ 1000 mL on intraoperative evaluation. With a median follow-up of 48 months, the median OS after ovarian metastasectomy for all patients was 8.2 months (95% CI 7.2-9.3 months). In univariate analyses, there is significant (8.0 months vs. 41.0 months, P = 0.000) difference in OS between patients with gastrointestinal cancer origin from breast origin, and between patients with gastric origin from colorectal origin (7.4 months vs. 8.8 months, P = 0.036). In univariate analyses, synchronous metastases, locally invasion, massive intraoperative ascites (≥ 1000 mL), and combined metastasis, were identified as significant poor prognostic factors. In multivariate analyses combined metastasis (RR, 1.72; 95% CI, 1.09-2.69, P = 0.018), locally invasion (RR, 1.62; 95% CI, 1.03-2.54, P = 0.038) and massive intraoperative ascites (RR, 1.58; 95% CI, 1.02-2.49, P = 0.04) were independent factors for predicting unfavorable overall survival.

**Conclusion:**

Ovarian metastases are more commonly originated from primary gastrointestinal tract. The prognosis of ovarian metastasis is dismal and the benefit of ovarian metastatectomy is limited. Combined metastasis outside ovaries, locally invasion and massive intraoperative ascites were independent factors for predicting unfavorable overall survival. The identification of the primary tumor is required to plan for adequate treatment for this group of patients.

## Background

The differential diagnosis of an ovarian mass is complex as approximately 7% of ovarian masses encountered clinically are metastatic lesions, the most common sites of origin being the gastrointestinal tract and breast. Often time surgical exploration is necessary to arrive at the correct diagnosis. The diagnosis and management of patients with ovarian metastastic extragenital primary cancer are typically challenging where inaccurate diagnosis has been reported in as many as 50% of the cases even with thorough preoperative work-up due to the presenting atypical symptoms [1-4]. Unlike the management of primary ovarian cancer including maximum tumor volume reduction which has demonstrated survival benefit [5,6] and CA125 as a predictive factor for tumor recurrence and treatment response, the value of similar surgical approach and post-surgery CA125 monitoring for metastatic ovarian tumor is uncertain [7]. As the biological behavior and clinical outcome of the ovarian metastatic lesions from extragenital cancer origins are rarely summarized, we presented in this study the characteristics and outcomes for all patients with surgically confirmed ovarian metastases from extragenital primary cancer in a single institution during an 8-year period.

## Methods

Patients who had undergone ovarian resection and diagnosed with ovarian metastases from extragenital tumor between April 2003 and May 2011 were identified retrospectively from the database of the Department of Pathology at Fudan University Shanghai Cancer Center (FUSCC). Chart review was performed for all cases for verification of the availability of complete post-surgery report, pathology report with immunohistochemistry staining, complete treatment history for the primary cancer at our institute. 147 cases who met the above criteria were included in our analysis. Clinical and pathological variables including preoperative CA125, CEA and LDH levels, intraoperative evaluation, size of ovarian metastases of pathologic gross specimen, pathology reports, primary tumor site, surgical procedures, and subsequent therapy at the department of medical oncology for the primary cancer were collected. Overall survival (OS) was calculated from the date of ovarian metastatectomy to death or last follow-up time. In patients whose time interval between the diagnosis of the primary tumor and that of the ovarian metastasis exceeded 6 months were defined as metachronous metastasis in current study.

Written informed consent for the use of patient information in research analysis was obtained from all patients at the time of admission as a routine practice at FUSCC. The study had been approved by the FUSCC Ethic Committee for Clinical Investigation.

### Statistical analysis

Survival analysis was carried out using life-table and Kaplan-Meier method. Actuarial curves were compared by the two-tailed log-rank test with a statistical significance level of 0.05. The independent prognostic significance of variables on the survival, proved to be significant factor in univariate analysis, was tested in proportional hazards regression models described by Cox. The estimates of the models are given as hazard ratio (HR) with 95% confidence intervals (95% CI).

## Results

### Characteristics of ovarian metastases

*Clinical characteristics* All patients’ characteristics were listed in Table 1. The clinical characteristics of the 147 patients included in the analysis were listed in Table 1. Patients were predominantly young with a median age at diagnosis of 47 years old (range: 21-78years), 81 (55.1%) being premenopausal. The most common primary cancer were colorectal (72, 48.9%), gastric (60, 40.8%), and breast (12, 8.2%). There were more frequent colon (54/72, 75%) origins compared to that of rectal (18/72, 25%). The majority of the cases (105, 71.4%) presented high pre-operation serum CA125 level, 51 (34.7%) exhibited high pre-operation serum CEA level and 28 (19%) patients had increased pre-operation serum LDH level.

**Table 1:** Patient Characteristics and clinical variables

	**Patients**	
	**N**	**%**
*Age (years)*		
Median (range)	47 (21-78)	
*Menopausal stastus*		
Premenopausal	81	55.1
Postmenopausal	66	44.9
*Primary cancer site*		
Colon & Rectum	72	49.0
Rectum	18	25.0
Colon	54	75.0
Stomach	60	40.8
Breast	12	8.2
Biliary Duct	2	1.4
Liver	1	0.7
*Timing of metastasis to ovary*		
Metachronous	92	62.6
Synchronous	55	37.4
*Combined with metastases outside ovaries*		
No	107	72.8
Yes	40	27.2
*Ovary involvement*		
Bilaterial	97	66
Unilaterial	50	34
*Locally invasion*		
No	104	70.7
Yes	43	29.3
*Ascites*		
≥1000 mL	39	26.5
<1000 mL	108	73.5
*Size of ovarian metastases*		
Median (range)	9.0 (2.5-23cm)	
≤5 cm	45	30.6
5-10 cm	48	32.7
>10 cm	54	36.7
*Histologic type*		
Adenocarcinoma	91	61.9
Mucinous carcinoma	17	11.6
Signet ring cell carcinoma	39	26.5
*Pre-operation serum CA125*		
Normal	42	28.6
Elevated	105	71.4
*Pre-operation serum CEA*		
Normal	96	65.3
Elevated	51	34.7
*Pre-operation serum LDH*		
Normal	117	79.6
Elevated	28	6.0
Unknown	2	1.4

Among the 55 patients who had synchronous metastasis, thirty-three patients (33/55, 60%) underwent synchronous (16, 48.5%) or staged (17, 51.5%) primary tumor resection with ovarian metastasectomy, and the other 22 patients (22/55, 40%) received cytoreductive surgery along with primary tumor resection because of the presence of disease other than the ovaries. 92 patients ( 62.6%) had metachronous ovarian metastasis, with a median time to metastasis of 15 months (range: 7-203 months); the cumulative proportions of time to metastasis within 1-year, 2-year and 3-year were 30.4%, 66.3%, 79.3%, respectively. Among the patients with metachronous metastases, seventy-four patients (74/92, 80.4%) underwent total resection of the metastatic lesions, and the other 18 patients (18/92, 19.6%) palliative oophorectomy.

One hundred and seven (72.8%) patients had ovarian metastasis only while 40 (27.2%) patients had combined metastases outside the ovaries, including liver, lung, bone, lymph nodes and other distant organs. Among the 40 patients with combined metastases, 12 (30%) were admitted with initial diagnosis of ovarian cancer for tumor cytoreduction which turned to be gastrointestinal tract or breast cancer originated; 17 (42.5%) were evaluated to be resectable prior to surgery but extensive abdominal and pelvic metastases were found during the operation; palliative resection was conducted in 3 patients (7.5%) to relieve massive tumor compression. The remaining 8 patients (20%) received the surgery due to other unknown individual reasons.

*Pathological characteristics* Bilateral metastasis was found in 97 (66%) patients. The median ovarian tumor size was 9 cm (range: 2.5-23 cm); with 36.7% patients >10cm. Thirty-nine patients (26.5%) were noted to have massive intraoperative ascites ≥ 1000 mL. Forty-four (29.9%) patients presented with metastatic disease confined in the ovaries, while 103 (70.1%) varying degrees of local invasion adjacent to the ovaries (including mesentery, intestinal tube, peritoneum, omentum and Douglas pouch). There were ninety-one (61.9%) adenocarcinoma, 39 (26.5%) signet ring cell carcinoma among which 36 (92.3%) were gastric origin, 17 (11.6%) mucinous carcinoma among which 14 (82.4%) were colorectal cancer origin.

### Prognosis of ovarian metastasectomy

**Figure 1:**
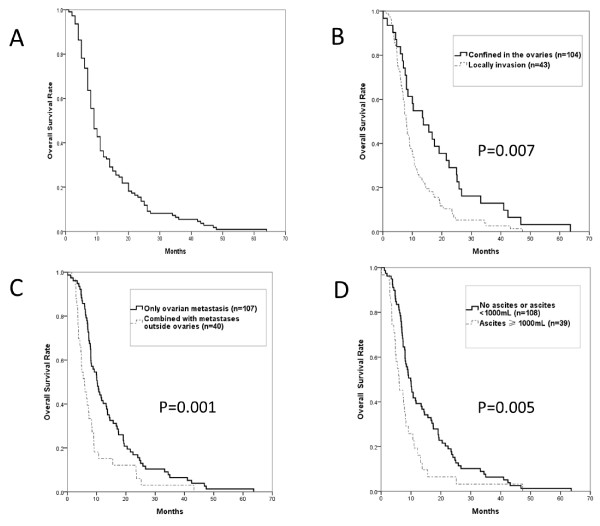
**Overall survival and subgroup analysis in patients with ovarian metastasectomy from extragenital primary sites.A** Overall survival time, **B** Locally invasion, **C** Combined Metastases, **D** Massive ascites ≥ 1000 mL.

With a median follow-up time of 48 months (range 3-97 months), 110 patients (74.8%) died and 37 patients (25.2%) were still alive. The median OS after ovarian metastasectomy from extragenital primary cancer was 8.2 months (95% CI 7.2-9.3 months, Figure 1A).

In univariate analyses (Table 2), extragenital primary cancer site from gastrointestinal cancer (vs breast cancer), gastric cancer (vs colorectal cancer), synchronus metastases (vs metachronus), locally invasion ( vs none) combined metastases (vs ovarian only), were identified as significant poor prognostic factors, as shown in Table 2 and illustrated in Figure 1B to C. No correlation was found between the numbers of positive locally invasion sites (mesentery, intestinal tube, peritoneum, omentum and Douglas pouch) and survival. Ovarian metastases originated from gastrointestinal tract cancer, mucinous carcinoma (N = 38)and signet ring cell carcinoma(N = 17)seemed to be the pathological type that carried a poorer prognosis, exhibiting a shorter survival trend with a median OS of 7.6 mo, compared to that of adenocarcinoma cancer(N = 76)of 8.5 mo (P = 0.076, data not shown in Table 2).

**Table 2:** Univariate analysis for survival of ovarian metastases from extragenital cancer

	**Patients %**	**Median OS (m)**	**95% CI (m)**	** *P* value**
*Primary cancer site*				
Colon & Rectum	49.0	8.8	6.5-11.2	0.036†
Stomach	40.8	7.4	6.3-8.4	
Breast	8.2	41.0	2.0-85.0	0.000‡
Biliary Duct	1.4	6.0		
Liver	0.7	NR		
*Timing of metastasis to ovary*				
Metachronous	62.6	10.2	8.3-13.2	0.027
Synchronous	37.4	8.1	6.7-9.4	
*Combined with metastases outside ovaries*				
No	72.8	10.2	8.1-12.4	0.001
Yes	27.2	5.9	3.8-8.0	
*Locally invasion*				
No	70.7	13.7	6.4-21.0	0.007
Yes	29.3	8.0	7.1-9.0	
*Ascites*				
≥1000 mL	26.5	6.0	8.2-11.7	0.005
<1000 mL	73.5	9.9	3.5-8.5	

†P value for colorectal cancer versus gastric cancer; ‡ P value for breast cancer versus gastrointestinal cancer.

Patients’ menopausal status, lateralality, size of the ovarian metastases, pre-operation serum CA125, CEA and LDH level were also analyzed in univariate analyses but these factors were not prognostic indicators for survival after the development of ovarian metastases (P > 0.05).

Using Cox regression, multivariate analyses demonstrated that combined metastasis (RR, 1.72; 95% CI, 1.09-2.69, P = 0.018), local invasion (RR, 1.62; 95% CI, 1.03-2.54, P = 0.038) and massive intraoperative ascites ≥ 1000 mL (RR, 1.58; 95% CI, 1.02-2.49, P = 0.04) were independent factors for predicting unfavorable overall survival (Table 3).

**Table 3:** Multivariate analysis for survival

	** *P* value**	**Relative Risk**	**95% CI for Exp. B**	
			**lower**	**upper**
combined metastasis	0.018	1.72	1.095	2.688
Locally invasion	0.038	1.62	1.028	2.544
massive ascites ≥ 1000 mL	0.04	1.58	1.02	2.495
Primary cancer site	0.068	0.77	0.59	1.02
Timing of metastasis to ovary	0.543	1.16	0.72	1.88
Histologic type	0.51	1.20	0.81	1.59

## Discussion

The outcome and prognosis of 147 patients with surgically confirmed ovarian metastases from extragenital primary cancer in our series reflected the complexity and challenges for the management of this specific population. 12 patients with initial preoperative diagnosis of primary ovarian cancer turned out to have ovarian metastases from gastrointestinal tract or breast cancer. The prognosis of ovarian metastases is dismal and the benefit of ovarian metastatectomy remained to be elucidated.

The mechanism of ovarian metastasis has largely been discussed in the literatures. Besides the commonly described routes of metastases for metastastic ovarian tumors arising from extragenital primary cancer that include direct invasion, surface implantation, other possible route for gastric cancer to disseminate to ovary may include lymphatic drainage via the receptaculum chili to the urogenital lymph vessel trunks, and, hematogenous spread from gastrointestinal tract tumors [8]. The lymphatic dissemination and transcoelomic spread are also proposed to be important mechanisms due to the high incidences of synchronized involvement of peritoneum and lymph nodes in colorectal cancer [9]. The exact mechanism of the spread of breast cancer to the ovaries had not been elucidated but the risk of primary ovarian cancer is increased in women with breast-ovarian cancer syndrome (BRCA1/2 mutation) [10], lymphoma had been reported to spread to the ovaries but there was none in our series [4].

Recent observations have reported a higher incidence of colorectal origin compared to gastric origin, and more frequently from colon rather than rectum [2,4,11-13]. Similar phenomenon was observed in our study. Nearly half of our patients had primary cancer that arose from colorectal cancer, and the ratio of colon to rectum was 3:1. Radiotherapy for rectal cancer with T3/4 or positive lymph node disease may be the contributing factor as ovarian micrometastases are eradicated and ovarian blood supply is impaired, reducing the risk of ovarian spread. Colonoscopy should not be omitted in cases presenting with ovarian tumors as evident colorectal cancer-related symptoms that may include defecate change or rectal bleeding were rarely the initial presentation [14,15].

The survival of the ovarian metastases patients from extragenital tumors is related to the primary tumor sites. In our series, patients with tumors originated from breast exhibited the best median survival time of 41 months, followed by those of colorectal and stomach, with the median survival time of 8.8 months and 7.4 months, respectively, which is comparable with previous data [16]. This result may be partly attributable to the nature of breast cancer having a better prognosis compared to the tumor from GI tract. Differences in tumor biology among individual patients, tumor types, or even within a given tumor, may also contribute to survival. As is previously reported [12-17], the median survival among patients with ovarian metastases of the gastrointestinal cancer origin was 13-30 months. The estimated 5-year disease free survival of the patients without other metastastic lesions reached 40% after complete resection [13]. However, the survival of the patients with gastrointestinal origin in our series was disappointing, with the median OS of 8.2 months. One explanation could be due to the fact that nearly 40% of the patients had synchronous ovarian metastases, representing a patient population of poorer prognosis. Moreover, the proportion of pathological type including mucinous carcinoma and signet ring cell carcinoma was nearly 40% in this cohort, which was also an unfavorable survival factor. Another reason could be that 30% of the patients were presented with combined extensive metastases at the diagnosis, indicating worse prognosis as found in other studies [13,15,16].. Peritoneal dissemination was reported as an adverse factor influencing the survival time [15]. In our analysis, huge volume of ascites and high incidence of local invasion were also determined as a poor prognostic factor. Additionally, the finding that there was no survival difference based on tumor size and laterality indicates that the development of ovarian metastases is a sign of a more aggressive disease and the ovarian metastases are diagnosed late in the cancer disease process.

Therefore, whether maximal surgical debulking should be conducted was controversial. Aggressive therapeutic measures similar to the practice for primary ovarian cancer had been advocated, especially in the case of colorectal cancer with ovarian metastases which was less responsive to chemotherapy [18,19]. Bilateral oophorectomy for ovarian metastasis from colorectal cancer have been shown to have a positive impact on disease-free and overall survival in isolated ovarian metastases patients in an Italian study [20]. Also, for patients with gastric cancer, a Korean study suggested that debulking or gastrectomy plus metastasectomy may bring survival benefits for patients with distant metastases who were receiving systemic chemotherapy [21]. However, other believed that metastatectomy should be reserved for curative intent because ovarian metastases tends to spread via a transcoelomic route and hence, a harbinger of peritoneal metastasis [9,22,23]. It remains unclear whether prophylactic bilateral oophorectomy would improve the survival, and routine practice of prophylactic bilateral oophorectomy may not be justified as to the low frequency of isolated ovarian involvement without distant metastases [9]. The effect of metastatectomy could not be determined in this study with its small sample size and retrospective in nature. Variability of distant metastatic status besides the ovaries and surgical procedures would bias the evaluation of surgery-related quality of life and long-term survival. In our study, it appeared that palliative surgery did not improve the long-term survival and well-designed prospective trials focusing on this aspect is needed to resolved the issue.

Effective methods have to be introduced, as traditional tumor marker in ovarian tumor, CA125, was not specific in predicting the survival of patients with ovarian metastases from extragenital cancer. PET/CT may be considered as an alternative approach in search for primary tumor sites and it was reported that a variable maximum SUV in ovarian metastases may be correlated to different primary origins [24].

## Conclusion

Ovarian metastases are more commonly seen to originate from primary gastrointestinal tract in our study. The prognosis of ovarian metastasis is dismal and the benefit of ovarian metastatectomy is limited. Those with combined metastasis outside ovaries, locally invasion and massive intraoperative ascites were independent factors for predicting unfavorable overall survival. The identification of the primary tumor is required to plan for adequate treatment for this group of patients. Image scanning and gastrointestinal endoscopy should be recommended before the ovarian metastatectomy.

## Competing interests

The authors, their immediate families of this paper have no potential conflict of interest to disclose.

## Authors’ contributions

WL participated in acquisition of data, analysis and interpretation of data, and drafting of the manuscript. HW participated in the ovarian operation and acquisition of data. JW participated in pathology review. FL and XZ participated in acquisition of data. ZW conceived of the study, participated in its design and coordination and revised the final manuscript. All authors have read and approved the final manuscript.

## Pre-publication history

The pre-publication history for this paper can be accessed here:

http://www.biomedcentral.com/1471-2407/12/278/prepub
